# Enteric pathogens and factors associated with acute bloody diarrhoea, Kenya

**DOI:** 10.1186/s12879-016-1814-6

**Published:** 2016-09-06

**Authors:** Charles Njuguna, Ian Njeru, Elizabeth Mgamb, Daniel Langat, Anselimo Makokha, Dismas Ongore, Evan Mathenge, Samuel Kariuki

**Affiliations:** 1 Jomo Kenyatta University of Agriculture and Technology, Nairobi, Kenya; 2 World Health Organization, Nairobi, Kenya; 3 Kenya Medical Research Institute, Nairobi, Kenya; 4 Ministry of Health, Nairobi, Kenya; 5 University of Nairobi, Nairobi, Kenya

**Keywords:** Case, Control, Acute bloody diarrhoea, Nairobi, Kilifi, Factors, Enteric pathogens

## Abstract

**Background:**

Shigellosis is the major cause of bloody diarrhoea worldwide and is endemic in most developing countries. In Kenya, bloody diarrhoea is reported weekly as part of priority diseases under Integrated Disease Surveillance and Response System (IDSR) in the Ministry of Health.

**Methods:**

We conducted a case control study with 805 participants (284 cases and 521 controls) between January and December 2012 in Kilifi and Nairobi Counties. Kilifi County is largely a rural population whereas Nairobi County is largely urban. A case was defined as a person of any age who presented to outpatient clinic with acute diarrhoea with visible blood in the stool in six selected health facilities in the two counties within the study period. A control was defined as a healthy person of similar age group and sex with the case and lived in the neighbourhood of the case.

**Results:**

The main presenting clinical features for bloody diarrhoea cases were; abdominal pain (69 %), mucous in stool (61 %), abdominal discomfort (54 %) and anorexia (50 %). Pathogen isolation rate was 40.5 % with bacterial and protozoal pathogens accounting for 28.2 % and 12.3 % respectively. *Shigella was* the most prevalent bacterial pathogen isolated in 23.6 % of the cases while *Entamoeba histolytica* was the most prevalent protozoal pathogen isolated in 10.2 % of the cases. On binary logistic regression, three variables were found to be independently and significantly associated with acute bloody diarrhoea at 5 % significance level; storage of drinking water separate from water for other use (OR = 0.41, 95 % CI 0.20–0.87, *p* = 0.021), washing hands after last defecation (OR = 0.24, 95 % CI 0.08–.076, *p* = 0.015) and presence of coliforms in main source water (OR = 2.56, CI 1.21–5.4, *p* = 0.014). Rainfall and temperature had strong positive correlation with bloody diarrhoea.

**Conclusion:**

The main etiologic agents for bloody diarrhoea were *Shigella* and *E. histolytica*. Good personal hygiene practices such as washing hands after defecation and storing drinking water separate from water for other use were found to be the key protective factors for the disease while presence of coliform in main water source was found to be a risk factor. Implementation of water, sanitation and hygiene (WASH) interventions is therefore key in prevention and control of bloody diarrhoea.

## Background

Globally, there are nearly 1.7 billion cases of diarrhoeal diseases every year with around 800,000 deaths among children under five year [1]. Shigellosis is a major cause of diarrhoea-related morbidity and mortality, especially in developing countries, with an estimated annual incidence of 165 million cases and 1 million deaths [2, 3]. Ninety-nine percent of infections caused by *Shigella* occur in developing countries [4]. Kenya has been experiencing a significant increase in acute bloody diarrhoea cases especially in Coast, Western, Nyanza and Nairobi regions. The cases reported through the weekly Integrated Disease Surveillance and Response System (IDSR) increased from 48,272 in 2009 to 64,107 in 2010. Despite an increase in the number of cases, the role of the laboratory in confirmatory diagnosis has been limited due to its weak capacity hence antimicrobial treatment of most patients is based on clinical diagnosis upon first contact in the health facility. Identifying the risk factors associated with the disease will provide a platform for putting in place mitigation measures that will reduce the morbidity and mortality rates attributed to acute bloody diarrhoea.

It has been demonstrated through studies that the incidence of acute bloody diarrhoea and other enteric infections are affected by rainfall and temperature variations [5–10]. Information on seasonality of the disease is key to strengthening the early warning system, prevention and control. In Kenya, no studies have been contacted to demonstrate any correlation between acute bloody diarrhoea and the key climatic drivers.

The objectives of the study were to: identify the presenting clinical features of acute bloody diarrhoea, determine the prevalence and distribution of enteric pathogens isolated from stool specimens of patients with acute bloody diarrhoea, demonstrate any existing differences in seasonality of acute bloody diarrhoea between urban and rural populations and to determine the factors associated with acute bloody diarrhoea.

## Methods

### Study site

The study was conducted in Kilifi County Hospital, Bamba Sub County Hospital and Vipingo Health Centre in Kilifi Sub-County and in African Medical Research Foundation (AMREF) Kibera Health Centre, Lang’ata Health Centre and Riruta Health Centre in Nairobi West Sub-County. The two Sub-Counties and the six health facilities were selected because they had high incidences of acute bloody diarrhoea in 2009–2010. Kilifi Sub-County is one of the 6 Sub-Counties in Kilifi County. The population of Kilifi Sub-County was 456,297 (2009 Census) and it is a largely rural population. The Sub-County has a total of 73 health facilities but accessibility of health services is low; 57 % of the populations live over 5kms from the nearest health facility (Kilifi District Strategic Plan, 2005–2010). Nairobi West is one of the three Sub-Counties in Nairobi County. The population of Nairobi West was 684,765 (2009 Census) and is largely urban. Kibera slum; one of the largest slums in Africa is located in Nairobi West Sub-County.

### Study design

A hospital based matched case control study was conducted with neighbourhood controls between January and December 2012. The ratio of cases to controls was 1:2 and the calculated minimum sample size was 198 cases and 396.

### Enrolment of cases and controls

A case was defined as a person of any age who attended outpatient clinic at the selected health facilities in Kilifi and Nairobi West Sub-Counties between January and December 2012 with acute diarrhoea with visible blood in the stool. Cases with concomitant infection and those with persistent diarrhoea (lasting ≥14 days) were excluded from the study. The cases were enrolled at the outpatient department of the 6 selected health facilities. Within 14 days of enrolment of a case, community health workers visited cases at their homes. A case was matched by age group to two healthy controls (<2 years, 2–4 years, 5–10 year, 11–17 years, 18–65 and >65 years). A control was defined as a healthy person of similar age group and sex with the case and lived in the neighborhood of the case. Any control person who reported any form of diarrhoea or any other gastrointestinal symptoms within 14 days of enrolment was excluded from the study. To identify the controls, the interviewers first identified a case’s home then selected two households in the neighborhood using systematic random sampling. In each of the households, one eligible control was randomly selected.

### Specimen collection

Stool samples were collected from all the cases at the health facility level. To ascertain that the controls were healthy, stool samples were also collected from 25 % of the controls at the household level. The samples were delivered to the laboratory within 2 h. Where the stool specimen could not reach the laboratory within two hours, a Cary-Blair transport medium was used.

Water samples were collected from the homes of all cases and 25 % of the controls. A total of two samples were collected, one from domestic sources e.g. borehole, river, stream, well, tap and the other from household container (drinking). The samples were transported to laboratory with 2 h of collection.

### Data collection

Data was collected using four different tools; case report form, laboratory based surveillance form, household investigation form and water sample collection form. The case report form was filled for all the cases. The information collected included: demographic data, vital signs, signs and symptoms, disease history and treatment information. The household investigation form was used to collect data on demographics as well as risk factor information which were classified into four broad categories as follows; water safety, food safety, hygiene and environmental sanitation. The laboratory based surveillance form and the water sample collection forms accompanied the stool and water samples respectively.

Two sets of data on rainfall and temperature for Kilifi and Nairobi counties were obtained from the Kenya Meteorological Department; the mean monthly rainfall and temperature for 2012 and the long term mean rainfall and temperature (1971–2012). The mean monthly rainfall and temperature for the year 2012 (study period) were compared with the number of cases of acute bloody diarrhoea of each month. Pearson’s correlation was used to establish the presence and strength of association.

### Laboratory analysis of stool samples

Laboratory tests were done in the health facility and the National Public Health Reference Laboratories according to standard operating procedures. The methods included microscopy for erythrocytes and parasites as well as culture for bacterial enteric pathogens. Stool samples were examined macroscopically for gross blood and mucus. Wet mounts of fresh stool were made in normal saline and examined for parasites and erythrocytes. Direct microscopic examination of fresh stool was used to detect *Entamoeba histolytica, Schistosoma mansoni, Giardia lamblia* and *Trichuris trichiura*. Stool swabs and specimens were inoculated onto the surface of MacConkey agar (MAC) and eosin methylene blue agars and streaked for colony isolation. At the national reference laboratory, the culture plates were incubated at 37 °C for 24 h, the non-lactose fermenting colonies (NLFs) were picked and subjected to a gram stain. Subsequently, all the gram-ve colonies were picked from the respective plates and prepared for biochemical identification using the semi-automated bacterial identification systems, the Vitek 2®. The colonies were briefly emulsified into 0.45 % normal saline solution for the system to attain a 0.5–0.63 McFarland strength. The gram -ve identification cassette was inserted into the respective tubes and then into the system. After 18–24 h of incubation the biochemical reactions were obtained through a print-out from the machine.

The results of bacterial cultures were shared with health facility staff at each surveillance site. Every patient with positive stool culture results, a reassessment of their clinical condition, either by telephone contact or follow up visit, was arranged. Any change in management was recorded in the case notes. Pathotyping *E.coli* isolates to screen for the five types of diarrhoeagenic E.coli (enteropathogenic *E. coli* (EPEC), enterotoxigenic *E.coli* (ETEC), enteroinvasive *E. coli* (EIEC), enteroaggregative *E. coli* (EAEC), and enterohemorrhagic *E. coli* (EHEC) was done using the real time Polymerase Chain Reaction (PCR) protocol and primers earlier described by Hardegen [11]. The PCR mix contained; 2× quantitect probe PCR mastermix, 0.3 μl forward primer (40 μM), 0.3 μl reverse primer (40 μM), 0.15 ul probe (40 μM) and 12.25 μl nuclease free water. Target specific master mixes were made, 28 μl transferred into 0.1 ml PCR tubes and 2ul of the extracted Deoxyribonucleic Acid (DNA) added into the specific tubes. The thermocycling conditions consisted of cycles of hot start activation at 95 °C for 15 min, amplification cycles of 95 °C, 15 s, 55 °C, 60 s. The amplification step was repeated for 45 cycles. Any sample with a cycle threshold value (ct) of <37 was considered positive. Any sample with a threshold of >37 were analyzed and considered as negative, positive or indeterminate based on the characteristics of the curve.

#### Bacteriological analysis for water samples

Each water sample was thoroughly mixed by shaking the container several times. The samples (10 mL) were diluted with double strength MacConkey broth in universal bottle. One bottle was incubated at 37°c in order to isolate non-faecal coliforms, while the other was incubated at 44 °C in order to isolate thermotolerant (faecal) coliforms. After 24 h incubation, positive cultures (evidence of turbidity or gas production) were sub-cultured onto agar and MacConkey agar plates. The bacterial isolates were further identified using their biochemical reactions on Analytical Profile Index (API) −20E strips. The isolates were kept frozen on protected beads at −70 °C until used.

## Results

We enrolled 805 participants (284 cases and 521 controls) into the study between January and December 2012. The targeted case: control ratio of 1:2 was 45 controls short of being achieved which represented a 91 % success rate. The mean age of the cases was 24.4 years with a range of 1 month to 73 years while the mean age of controls was 27.5 years with a range of 3 months to 73 years. The proportion of females was 56 % among cases and 67 % among controls. About 61 % of the cases and 63 % of the controls resided in the rural areas (Table 1).

**Table 1:** Demographic characteristics of the cases and controls, Jan – Dec 2012

Variable	Cases n (%)	Controls n (%)	Total n (%)
Gender			
Male	124 (42)	173 (33)	297 (37)
Female	160 (56)	346 (67)	506 (63)
Age Group			
<5 years	63 (22)	72 (14)	135 (17)
5- < 11 years	12 (4)	23 (5)	35 (4)
11- < 18 years	33 (12)	25 (5)	58 (7)
18-65 years	166 (59)	372 (74)	538 (69)
>65 years	7 (2)	9 (2)	16 (2)
Residence			
Kilifi	174 (61)	329 (63)	503 (62)
Nairobi West	110 (39)	192 (37)	302 (38)

**Fig. 1:**
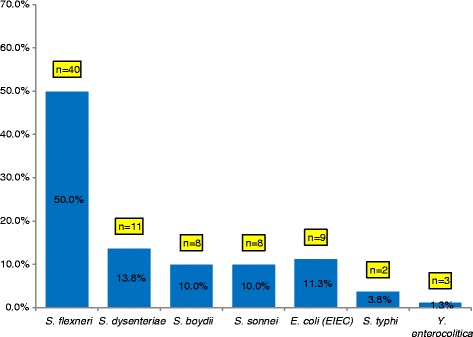
Distribution of bacteria isolates among patients with acute bloody diarrhoea in selected hospitals in Kilifi and Nairobi Counties

A total of 398 stool samples were collected; 284 from cases and 114 from controls. Enteric pathogens known to cause bloody diarrhoea were isolated in 115 (40.5 %) of the cases. The isolation rate among the rural population (Kilifi) was 24.7 % while among the urban population (Nairobi) it was 65.5 %. Bacterial pathogens were isolated in 80 (28.2 %) of the cases, the distribution was as follows; S*higella* species (83.8 %), *E.coli* (11.3 %), *Salmonella* species (3.8 %) and *Yersinia* enterocolitica (1.3 %) (Fig. 1). The most prevalent *Shigella* species isolated was *Shigella flexneri* (14.1 %) while *Shigella dysenteriae, S. boydii* and *S. sonnei* accounted for 3.9, 2.8 and 2.8 % respectively. Enteroinvasive *E.coli* was the only pathotype isolated among the cases accounting for 3.2 %. Protozoal pathogens were isolated in 12.3 % of the cases with trophozoites *of E. histolytica* and *G. lamblia* being isolated in 10.2 % and 1.4 % of the cases respectively (Table 2).

**Table 2:** Etiologic agents among patients with acute bloody diarrhoea in selected hospitals in Kilifi and Nairobi Counties

Lab Method	Kilifi		Nairobi		Total n (%)	
1. Direct Microscopy and concentration	Case *N* = 174 n (%)	Control *N* = 88 n (%)	Case *N* = 110 n (%)	Control *N* = 26 n (%)	Case *N* = 284 n (%)	Control *N* = 114 n (%)
Trophozoites (*n* = 398)						
*Entamoeba histolytica*	8 (4.6)	0 (0)	21 (19.1)	1 (3.8)	29 (10.2)	1 (0.8)
*Giardia lamblia*	0 (0)	0 (0)	4 (3.6)	1 (3.8)	4 (1.4)	1 (0.8)
*Trichomonas hominis*	1 (0.6)	0 (0)	1 (0.9)	0 (0)	2 (0.7)	0 (0)
*E. histolytica & G. lamblia*	0 (0)	0 (0)	2 (1.8)	0 (0)	2 (0.7)	0 (0)
Cysts (*n* = 398)						
*Entamoeba histolytica*	25 (14.4)	1 (1.1)	14 (12.7)	1 (3.8)	39 (13.7)	2 (1.7)
*E. coli*	6 (3.4)	3 (3.4)	1 (0.9)	0 (0)	7 (2.5)	3 (2.6)
*Giardia lamblia*	0 (0)	0 (0)	4 (3.6)	2 (7.7)	4 (1.4)	2 (1.7)
*G. lamblia & E. histolytica*	0 (0)	0 (0)	1 (0.9)	0 (0)	1 (0.4)	0 (0)
Ova (*n* = 398)						
*Trichuris trichuria*	0 (0)	0 (0	0 (0)	1 (3.8)	0 (0)	1 (0.8)
*Ascaris lumbricoides*	0 (0)	0 (0)	1 (0.9)	1 (3.8)	1 (0.4)	1 (0.8)
2. Stool culture						
*Shigella*	33 (20)	0 (0)	33 (0.3)	1 (3.8)	67 (23.6)	1 (0.8)
*E. coli*	1 (0.6)	0 (0)	8 (7.2)	0 (0)	9 (3.2)	0 (0)
*Salmonella* spp	1 (0.6)	0 (0)	2 (1.8)	0 (0)	3 (1.1)	0 (0)
*Yersinia enterocolitica*	0 (0)	0 (0)	1 (0.9)	0 (0)	1 (0.35)	0 (0)

Apart from bloody diarrhoea, the other associated signs and symptoms were: abdominal pain (69 %), mucous in stool (61 %) abdominal discomfort (54 %) and anorexia (50 %). Fever was present in 89 (34 %) of the cases. The mean duration of diarrhoea among those in whom *Shigella* spp was isolated was 2.1 days with a median of 2 (interquartile range 2–3) while those in whom *E. histolytica* was isolated had a mean duration of diarrhoea of 1.9 days with a median of 2 (interquartile range 2–2.75). On comparing the differences in the two means using the *t*-test, there was no statistically significant difference between the two means (*p* = 0.65).

Drinking water in the sampled communities was found to be of variable microbial safety and quality. Most probable number (MPN) test was done to detect the coliform in water samples collected from households and main water sources. The microbial indicators of faecal contamination (total coliforms and faecal coliforms) were detected in household water and main source. About 27.8 % of the household water tested contained total coliforms and 14 % faecal coliforms whereas 25.1 % of the main source contained total coliforms and 9.2 % faecal coliforms.

**Fig. 2:**
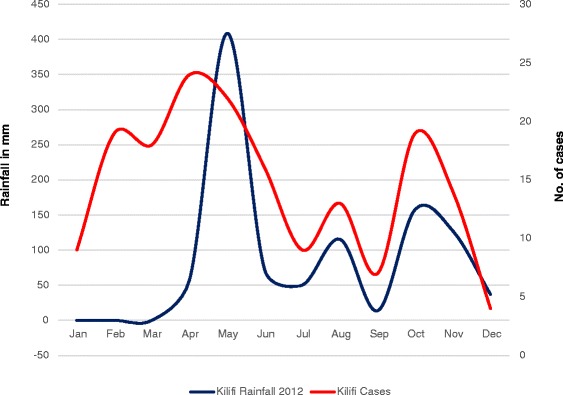
Correlation between acute bloody diarrhoea and mean monthly rainfall (2012) in Kilifi County

**Fig. 3:**
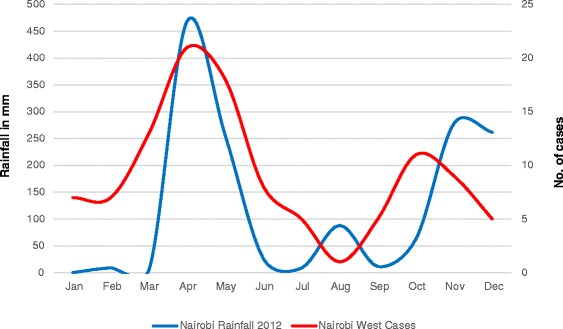
Correlation between acute bloody diarrhoea and mean monthly rainfall (2012) in Nairobi County

Climatic conditions differed in the two areas of study. In Nairobi, the average monthly rainfall during the study period was 123 mm, this was higher than the long term mean 85.2 mm for the period 1971–2012. In Kilifi, the average monthly rainfall for the study period was 86.6 mm which was lower than the long term mean of 86.9 mm. In Nairobi, the mean monthly maximum and minimum temperatures over the study period were 24.4 °C and 13.6 °C respectively. These were both higher than the long term mean maximum and minimum temperatures which were 24 °C and 13.2 °C respectively. In Kilifi, the mean maximum temperature for the study period was 29.9 °C which was similar to the long term mean of 29.8 °C. The mean minimum temperature for the study period was (24.0 °C), this was higher than the long term mean (23.4 °C). The seasonal patterns of acute bloody diarrhoea were the same in the two sites with two peaks in the year (April and October) (Figs. 2 and 3).

There was a positive correlation between acute bloody diarrhoea and the 2012 monthly mean rainfall both in rural and urban populations. In Kilifi Sub- county (rural), the correlation was moderate, Pearson’s *r* = 0.44 (Fig. 2) whereas in Nairobi West sub county (urban), there was a strong correlation, Pearson’s *r* = 0.61 (Fig. 3). There was also a positive correlation between acute bloody diarrhoea and the 2012 monthly mean maximum and minimum temperatures. The correlation with minimum temperatures was much stronger than that with maximum temperatures. In Kilifi (rural) the correlation was moderate, Pearson’s *r* = 0.45 for the mean monthly minimum temperatures and weak for maximum temperatures (Pearson’s *r* = 0.24). In Nairobi (urban), the correlation with mean monthly minimum temperatures was strong, Pearson’s *r* = 0.74; the association with maximum temperatures was however weak, Pearson’s *r* = 0.18.

Bivariate analysis identified eight [8] factors that were significantly associated with acute bloody diarrhoea, six protective and two risk factors (Table 3). The significant factors were included in the multivariate analysis. After running binary logistic regression, storage of drinking water separate from water for other use (OR = 0.41, 95 % CI 0.20–0.87, *p* = 0.021), washing hands after last defecation (OR = 0.24, 95 % CI 0.08–.076, *p* = 0.015) and presence of coliform in main source water (OR = 2.56, CI 1.21–5.4, *p* = 0.014) remained independently and significantly associated with acute bloody diarrhoea at 5 % significance level (Table 4).

**Table 3:** Factors associated with acute bloody diarrhoea

Factors	Cases		Controls		Odds Ratio	95 % CI	*P*-value
	Yes n (%)	No n (%)	Yes n (%)	No n (%)			
Main water source treated	79 (42.5)	107 (57.5)	183 (47.8)	200 (52.2)	0.807	0.871–1.764	0.235
Drinking water covered	222 (90.6)	23 (9.4)	470 (93.6)	32 (6.4)	0.657	0.376–1.149	0.139
Drinking water stored in super drum	29 (10.2)	255 (89.8)	90 (17.3)	431 (82.7)	0.545	.0349–0.851	0.007^a^
Drinking water stored separately	124 (49.8)	125 (50.2)	290 (57.9)	211 (42.1)	0.722	0.532–0.979	0.036^a^
Coliform in household water	44 (29.3)	106 (70.7)	40 (26.3)	112 (73.7)	0.341	0.702–1.924	0.341
Coliform in main water source	43 (31.4)	94 (68.6)	26 (18.8)	112 (81.2)	1.971	1.127–3.446	0.016^a^
Main water source protected	131 (78.0)	37 (22.0)	135 (86.5)	21 (13.5)	0.551	0.306–0.991	0.045^a^
Eating raw foods/vegetables	230 (91.6)	21 (8.6)	475 (94.2)	29 (5.8)	0.669	0.373–1.198	0.174
Always reheat food before eating	127 (55.5)	102 (44.5)	286 (59.5)	195 (40.5)	0.849	0.618–1.167	0.313
Eating from outside home	120 (48.6)	127 (51.4)	208 (41.8)	290 (58.2)	1.317	0.969–1.790	0.078
Toilet present in the compound	174 (69)	78 (31)	358 (71.9)	140 (28.1)	0.872	0.627–1.215	0.419
Toilet clean and covered	224 (78.6)	61 (21.4)	408 (78.5)	112 (21.5)	1.008	0.709–1.433	0.964
Always hand-wash after defecating	133 (53.6)	115 (46.4)	324 (65.2)	173 (34.8)	0.618	0.453–0.842	0.002^a^
Washed hands after last defecation	174 (74.7)	59 (25.3)	403 (86.3)	64 (13.7)	0.468	0.315–0.696	0.0001^a^
Always wash hands after disposing child’s stool	105 (56.8)	80 (43.2)	253 (65.4)	134 (34.6)	0.698	0.486–0.995	0.046^a^
Always washing hands before food preparation	119 (54.1)	101 (45.9)	256 (59.3)	176 (50.7)	0.81	0.584–1.124	0.207
Always washing hands before eating	219 (87.3)	32 (12.7)	441 (89.1)	54 (10.9)	0.838	0.526–1.336	0.457
Poor compound cleanliness	48 (19.2)	202 (80.8)	59 (12.1)	429 (87.9)	1.728	1.139–2.619	0.009^a^
Entered livestock pen past 2 weeks	31 (16.1)	162 (83.9)	63 (15.1)	353 (84.9)	1.072	0.671–1.713	0.771
Contact with livestock drinking water in the past 2 weeks	21 (10)	189 (90)	33 (7.4)	413 (92.6)	1.391	0.784–2.468	0.258

^a^Statistically significant factors

**Table 4:** Binary logistic regression analysis of statistically significant factors

Factors	*p*-value	Odds ratio	95 % CI	
			Lower	Upper
Drinking water storage in super drum	0.145	0.511	0.208	1.259
Storage of drinking water separate from water for other use	0.021^a^	0.412	0.195	0.873
Hand-washed after defecating	0.972	1.018	0.375	2.76
Hand-washed after last defecation	0.015^a^	0.244	0.079	0.757
Hand-washed after child’s stool disposal	0.339	0.649	0.267	1.575
Poor compound cleanliness	0.97	1.02	0.353	2.948
Main water source protected	0.813	1.123	0.432	2.921
Coliforms present in main source water	0.014^a^	2.555	1.208	5.404

^a^Statistically significant factors. The reliability of the model was 66.1 %

## Discussion

The proportion of cases presenting with diverse clinical symptoms were lower in our study compared with the finding in a similar study in rural western part of Kenya where the main symptoms were reported as abdominal cramping (78 %), fever (76 %), nausea (53 %), vomiting (31 %), coincident mucous diarrhoea (82 %) and coincident watery diarrhoea (56 %) [12]. This variation could be attributed to improved health seeking behaviour among the cases and improvement in accessibility of health services over the years. There were no differences in the duration of diarrhoea among those with acute bloody diarrhoea due to bacterial and protozoal pathogens. The frequency of other clinical features were similar to a study done in Turkey [13].

The main etiologic agents for acute bloody diarrhoea were *Shigella* and *E. histolytica.* The *Shigella* isolation rate was approximately half of that seen in a study conducted in the western part of Kenya [12] but two-three times higher compared with other studies done in Iran and Cameroon [14, 15]. The proportions of the different species of *Shigella* isolated in our study showed a similar pattern to another study done in Kenya where *Shigella flexneri* was the commonest strain followed by *S. dysenteriae*, *S. boydii and S. sonnei* in decreasing order for both studies [12]. This was however contrary to the findings of other findings where *S. sonnei* was the most prevalent strain [14]. The isolation rate of *E. histolytica* is similar to that in a study in Nigeria which had an isolation rate of 11 % [16].

The study showed strong seasonal variation of acute bloody diarrhoea with highest number of cases from the month of April –June followed by October –December which corresponds to the long and short rainy seasons in Kenya. This results are consistent with a study done in United States of America (USA) where the trend of Shigellosis shows strong seasonal variations [17]. Our study indicates that rainfall and temperature could be the key climatic drivers in the transmission of acute bloody diarrhoea disease. In Nairobi, we detected a strong positive associations between the incidence of acute bloody diarrhoea and two climatic variables (rainfall and minimum temperature) while moderate positive associations were observed in Kilifi. There was a weak positive association between the incidence of the disease and maximum temperature in both sites. These results agree with a study done in China where the monthly incidence of dysentery was positively correlated with maximum temperature, minimum temperature and rainfall [10, 18]. The use of meteorological data for Malindi as a proxy for Kilifi could be a possible reason for the variation on the strength of the associations; the two coastal towns are 60 Kms apart. In addition, Nairobi is 426 Kms flight distance from Kilifi and has an elevation of 1795 m above the sea level compared to Kilifi which is at 0 m. These variations affect humidity and air pressure which have been found to have positively and negatively correlated with incidence of dysentery respectively [10, 19]. A study done in China indicated that a 1 °C increase in temperatures may cause more than a 12 % increase in the incidence of bacillary dysentery [6]. The impact of rainfall on diarrhoeal disease is however far from clear [10]. While the relationship between climate variation and diarrhoea diseases has received a great deal of attention recently [7, 10, 20], there are few papers examining the relationship in Africa, further studies are necessary in this area.

The transmission of dysentery could be affected by many factors, including people’s dietary pattern, hygiene behavior, susceptibility to different pathogen strains, and sensitivity to the available drugs as well as local weather conditions [9, 19].

Studies have indicated that hand washing, especially if soap is used, is effective in reducing substantially cases of dysentery, diarrhoea and secondary transmission [21–24]. Washing hands after defecation has been previously associated with decreased risk of *Shigella* infection [12]. In this study, washing hands after last defecation was found to be a significant protective factor against acute bloody diarrhoea. It has been estimated that the attributable risk for dysentery from not washing hands before preparing food in rural African communities is as high as 30 % [25]. In other studies, hand washing reduced the risks of severe intestinal infections and shigellosis by 48 and 59 % respectively [26] while improvements in hand hygiene resulted in reductions in gastrointestinal and respiratory illnesses by 31 % and 21 % respectively [24].

Interventions including promoting hand washing resulted in a 31 % reduction in diarrhoea episodes in communities in low-middle income countries [23]. In a study done in Karachi, Pakistan, households that received free soap and hand washing promotion for 9 months reported 51 % less diarrhoea than controls [22]. Washing hands with soap can reduce significantly the risk of diarrhoeal diseases in all age groups and interventions to promote hand washing might save a million lives in low-income areas where comparatively costly interventions, such as supply of safe water and improved sanitation are not possible [21, 23, 27]. More and better-designed trials are needed to measure the impact of washing hands on acute bloody diarrhoea in developing countries.

Safe water storage, treatment and hand hygiene have been shown to reduce fecal contamination and improve health. In our study, separating drinking water and water for others uses at household level was found to be protective. A study done in Peru, post-source contamination increased successively through the steps of usage from source water to the point of consumption where source water was microbiologically clean, but 28 % of 93 samples of water stored for cooking had fecal contamination [28]. Testing for evidence of water contamination has been traditionally accomplished by the detection or enumeration of total and fecal coliforms. Coliforms should not be detectable in treated water supplies, they can be used as an indicator of treatment effectiveness and to assess the cleanliness and integrity of distribution systems and the potential presence of biofilms [29, 30]. Coliform presence in main water source was an important finding in this study; communities continue to face challenges of accessing clean and safe water for domestic use. Consuming contaminated water with faecal coliforms increases the attributable risk for household members contracting water borne disease like dysentery, cholera, salmonellosis among others.

In the past decade, further evidence has emerged that supports the beneficial outcomes of water, sanitation, and hygiene interventions in developing countries [31]. A meta-analysis of the impact of such interventions concluded that increasing water quantity reduced the occurrence of diarrheal diseases by 25 %, whereas point-of-use (POU) household water treatment and improved sanitation led to reductions in diarrheal diseases of 35 and 32 %, respectively [32]. Sanitation and POU interventions may have resulted in greater reductions because they directly block pathways of exposure [32]. Other studies done in Kenya, Guatemala, and India have demonstrated that use of POU treatments leads to a reduction in diarrhoea by 40 % for PUR and solar disinfection and by up to 85 % for chlorine [22, 33, 34]. Use of chlorine may lead to a greater reduction in diarrhoea because of its advantages relating to low cost, ease of use, and its ability to be manufactured locally. However, continuous promotion on use of POU treatments is required among communities in order to realize the desired optimal levels for prevention of diarrhoea diseases. In a chlorine-disinfection and safe-storage project in rural Kenya, only 33 % of households had chlorine residual six months after implementation of the intervention, this was evidence of use of POU treatments [35].

Kenya has not yet achieved the targets of Millennium Development Goal 7. According to the United Nations indicators for 2015, the proportion of population using an improved drinking water source in rural and urban Kenya is at 57 and 82 % respectively [36]. An improved drinking-water source is defined as one that, by nature of its construction or through active intervention, is protected from outside contamination with faecal matter while an improved sanitation facility is defined as one that hygienically separates human excreta from human contact.

### Limitation of the study

Infection with Human Immunodeficiency Virus (HIV)/ Acquired Immunodeficiency Syndrome (HIVAIDS) has been found to be an important co-morbidity for bloody diarrhea [37, 38]. We were unable to ascertain the true HIV status of the enrolled study participants. The study was limited by our inability to culture campylobacter at the time of the study. Campylobacter was isolated in 7 % of patient’s presentation with bloody diarrhoea in Kenya [12]. The study was further limited in examining risk factors that may be unique for young children under 5 years who constituted about 17 % of our enrolled study participants.

## Conclusion

The main etiologic agents for acute bloody diarrhoea among communities in Kilifi and Nairobi Counties were *Shigella* and *E. histolytica*. The transmission of dysentery could be affected by many factors; in this study, good personal hygiene practices such as washing hands after defecation and storing drinking water separate from water for other use were found to be the key protective factors while presence of coliforms in main water source was found to be a risk factor. The prevailing local weather conditions (rainfall and temperature) were found to have a moderate-strong positive correlation with acute bloody diarrhoea. Promoting awareness of good personal hygiene, safe storage and treatment of water at the point of use are of great importance in prevention and control of the disease. These interventions could be effective in reducing the incidence substantially as well as secondary transmission.

## Abbreviations

CI: Confidence interval
HIV/AIDS: Human immunodeficiency virus (HIV)/ Acquired immunodeficiency syndrome
OR: Odds ratio
*P*: Probability value
PCR: Polymerase chain reaction
POU: Point-of-use
WHO: World Health Organization
